# The influence of a soil amendment on the abundance and interaction of arbuscular mycorrhizal fungi with arable soils and host winter wheat

**DOI:** 10.1099/acmi.0.000581.v5

**Published:** 2024-01-16

**Authors:** Thomas I. Wilkes

**Affiliations:** ^1^​ Department of Clinical, Pharmaceutical and Biological Science, School of Life and Medical Sciences, College Lane Campus, University of Hertfordshire, Hatfield, Hertfordshire AL10 9AB, UK

**Keywords:** arbuscular mycorrhizal fungi, compost, mycorrhizal abundance, soil management, winter wheat

## Abstract

Arbuscular mycorrhizal (AM) fungi have been shown to be associated with an estimated 70 % of vascular terrestrial plants. Such relationships have been shown to be sensitive to soil disturbance, for example, tillage in the preparation of a seed bed. From the application of arable soil management, AM fungal populations have been shown to be negatively impacted in abundance and diversity, reducing plant growth and development. The present study aims to utilise two sources (multipurpose compost and a commercial inocula) of mycorrhizal fungi for the amendment of arable soils supporting Zulu winter wheat under controlled conditions and quantify plant growth responses. A total of nine fields across three participating farms were sampled, each farm practicing either conventional, reduced, or zero tillage soil management exclusively. Soil textures were assessed for each sampled soil. Via the employment of AM fungal symbiosis quantification methods, AM fungi were compared between soil amendments and their effects on crop growth and development. The present study was able to quantify a mean 6 cm increase to crop height (*P*<0.001), 10 cm reduction to root length corresponding with a 2.45-fold increase in AM fungal arbuscular structures (*P*<0.001), a 1.15-fold increase in soil glomalin concentration corresponding to a 1.26-fold increase in soil carbon, and a 1.32-fold increase in the relative abundance of molecular identified AM fungal sequences for compost amended soils compared to control samples. Mycorrhizal inocula, however, saw no change to crop height or root length, AM fungal arbuscules were reduced by 1.43-fold, soil glomalin was additionally reduced by 1.55-fold corresponding to a reduction in soil carbon by 1.31-fold, and a reduction to relative AM fungal species abundance by 1.26-fold. The present study can conclude the addition of compost as an arable soil amendment is more beneficial for the restoration of AM fungi beneficial to wheat production and soil carbon compared to the addition of a commercial mycorrhizal inocula.

## Data availability statement

Much of the data is presented within the manuscript. Sequencing output files are uploaded to Figshare under doi: 10.6084/m9.figshare.22047464.

## Introduction

Arbuscular mycorrhizal (AM) fungi have existed in close symbiotic relations with terrestrial plants for an estimated 430 million years and are thought to have been the catalyst for such terrestrial plants to begin initial land colonisation [1]. One mechanism that may have contributed to the initial mutual symbiotic relationship is the acquisition and transfer of soil bound nutrients, typically not available to the plant without prior metabolism by the associated AM fungus [2]. Once acquired, nutrients are transported through the AM fungal mycelial network to intercellular root cortical arbuscules within the host’s root system [3]. AM fungal arbuscules are highly specialized structures with folded fungal and plant membranes, forming the peri arbuscular membrane, for increased surface area to facilitate nutrient transfer from AM fungus in exchange for plant-produced photosynthetic carbohydrates [4].

Due to their close symbiotic relationships with a host plant, AM fungi are extremely important to arable crop production. Several studies have shown the growth and development benefits from maintaining an undisturbed mycorrhizal soil population [5–7]. However, arable soil management practices employ a range of procedures to prepare a seedbed and/or remove emerging weeds prior to cultivating. Tillage is an example of soil-management practices employed for seedbed preparation [8, 9]. Conventional tillage (CT) can disturb and homogenize soils to a maximum depth of 30 cm, 20 cm in the UK, and the most damaging and disruptive tillage regime against AM fungal populations as well as well-developed hyphal networks [10, 11]. Through the disturbance of soil and its homogenization in CT, broken AM fungal hyphae and spores are distributed through a larger volume of soil, with the greatest abundance of AM fungi can usually be found within topsoils (<10 cm) of undisturbed soils [12]. Such a dilution effect reduces the ability and increases the time taken for AM fungi to propagate towards a new potential host and establish a symbiotic relationship. This is also seen from the increased time required for strigolactone, a plant-produced signalling molecule used to encourage AM fungal symbiosis, to diffuse through the greater volume of soil and trigger AM fungal growth [13–15]. However, zero tillage (ZT) is a stark contract to CT, having no soil disturbance characteristics. In ZT practices, seed is direct drilled through the previous crop’s residue [16]. Whilst ZT soil disturbance is minimal, the use of glyphosate as a herbicide introduces complications for topsoil AM fungal populations as explored by Wilkes *et al*. [17]. Whilst ZT is an example of conservation tillage, reduced tillage (RT) is another example. RT disturbs soil (up to 10 cm depth) to a lesser degree than CT. This partially preserves AM fungal populations [10, 12].

Several studies have used AM fungi as a soil inoculant in small scale plot and/or glasshouse manipulative experiments [18–21]. Several commercial products are available to introduce further AM fungal populations into soils to support the growth and development of several plant, and food-producing plant types. Berruti *et al*. [22] discuss the reviewed literature regarding the production of AM fungal inocula for large-scale field experiments, concluding that much of the literature favours a single AM fungal species to be used as an inoculant as opposed to multi-species inocula that have been shown to reduce the overall effectiveness of AM fungal inocula, reducing potential impacts on plant growth.

Therefore, to address tillage-reduced AM fungal populations and increase soil quality via mycorrhizal populations, the present study aims at investigating two types of soil amendment (compost and commercial mycorrhizal inocula) to increase AM fungal-host symbiosis in three types of tillage (conventional, reduced and zero) across four soil types (sand, sandy loam, clay, clay loam) supporting Zulu variety winter wheat, with focus on AM fungal abundance via characteristic growth attributes (root arbuscules, fungal biomass and soil glomalin) as proxy indicators as well as crop growth parameters (number of tiller, height of crop and root length). The present study will also comment on potential integration of these amendments into soil-management practices.

## Methods

### Field study

Farm records were obtained from three farms, two farms situated in central Hertfordshire and one in Dorset, UK. Records were used to determine soil type (sand, sandy loam, clay, clay loam – not all soil types were present at each sample site, therefore, not all tillage types covered the investigated soil types), crop rotation and crop type, fertilizer type and application rates for each field site (*n*=3 per farm) covering conventional (maximum soil inversion to 20 cm), reduced (maximum soil disturbance to 10 cm) and zero tillage (direct drilling practiced with minimal soil disturbance) soil-management practices. Sample sites were chosen for their similarity in fertilizer type and application rate, history of crop rotation, as well as to cover different soil types managed under the same tillage practice. A regular sampling grid of 50 m was constructed via QGIS (version 3.22.0) with 60 sample points per field. Sampling points within the grid were selected via a random number generator (*n*=10), with these points then used for all fields sampled. Each sample point was subject to the removal of 5 kg of topsoil (<10 cm depth from surface) in total. Soils, once sampled, were transferred into pots (see Experimental design) in the field without homogenizing soils in order to preserve mycorrhizal structures and to maintain the effects of applied tillage. Soil samples were tested for soil type, and nutrient content (nitrogen, potassium and phosphorous) to confirm farm-held records, as well as ergosterol and correlated glomalin to determine AM fungal biomass within sampled soils.

### Experimental design

A total mass of 500 g of each soil type per tillage was placed into a plastic pot (7 cm diameter × 9 cm height) for control (non-treated), compost amended, and commercial mycorrhizal inoculated treatments with 12 replicates for destructive sampling covering 12 weeks of growth (*n*=648, composed from three replicates per treatment per soil/tillage per week). In order to inoculate soils with the mycorrhizal inoculant, RootGrow (containing 6 AM fungal species: *Diversispora* spp.*, Claroideoglomus clarodeum, Funneliformis geosporus, Funneliformis mosseae, Glomus microaggregarum* and *Rhizophagus intraradices*) was utilised as a commercial source of mycorrhizal spores (500 spores per gram) and amended to soils following the ratio as determined by the manufacturer. Soils amended with compost received 50 g of J Arthur Bowers multipurpose compost following the removal of 50 g field sampled soils to maintain an overall mass of 500 g. Zulu variety winter wheat was used, sourced as farm saved seed, with a single seed placed in the centre of each potted soil. An initial 100 ml water was applied over the soil surface to each pot, with subsequent 50 ml water applied weekly for the duration of the study. Wheat was maintained in controlled growth conditions (18±2 °C, 37±3% relative humidity, 15,260 lux) (Weisstechnik PG4 plant growth chamber SGC120, MI, USA).

### Physiochemical properties of the soil

#### Soil texture

Soil texture was determined by the methodology described by Brown and Wherrett [23].

#### Soil nutrient testing

Soil nitrogen (N), phosphorus (P) and potassium (K) were monitored via photospectrometry, as described by Wilkes [24], throughout to maintain a consistent concertation of each nutrient over the 12 week growth period. Sodium nitrate, sodium phosphate and potassium chloride were used, respectively, if soil NPK concentrations needed to be adjusted in accordance with optimal soil NPK levels provided by Teagasc [25], Potash Development Association (PDA) [26] and Oldham [27].

#### Soil carbon via loss on ignition

A modified loss on ignition (LOI) method from Myrbo *et al*. [28] was adopted as follows; 5 g of dried soils of each soil and tillage type were weighted out into crucibles and left in a muffle furnace at 400 °C for 18 h. After sufficient time had elapsed, allowing the furnace to cool, samples were re-weighed and percentage difference was calculated.

### Ergosterol HPLC

Soil ergosterol levels were determined using a modified methodology originally developed by Millie-Lindblom *et al*. [29] as reported by Wilkes *et al*. [17, 30, 31]. In brief, a 1 g sub-sample of potted soils was freeze dried using a ChechaTech System (MechaTech, Bristol, UK) LSB40 freeze drier chamber, Edwards RV5 vacuum pump (Thermo Fisher Scientific, Loughborough, Leicestershire, UK) and MicroModulyo (Thermo Fisher Scientific, Loughborough, Leicestershire, UK) freeze drier. The duration of each cycle was 21 h. Of the freezer dried soil, 150 mg was weighed into 50 ml centrifuge tubes. To each centrifuge tube, 4 ml of 10 % KOH in methanol [methanol hydroxide (MeOH)] and 1 ml cyclohexane was added and placed in an ultrasonic water bath for 15 min at 15 °C and 40 KHz (Crenex, Thermo Fisher Scientific, Loughborough, Leicestershire, UK) before incubation at 70 °C for a maximum of 2 h. Samples were cooled to room temperature and 1 ml of Milli-Q water was added with a further 4 ml of cyclohexane, vortexed (FisherBrand, Thermo Fisher Scientific, Loughborough, Leicestershire, UK) at maximum speed for 60 s then centrifuged at 1000 ***g*** for 60 s (Sigma 1–14, SciQuip, Newtown, Wem, Shropshire, UK). The cyclohexane fraction was transferred to a clean test tube and all cyclohexane evaporated, before 1 ml of HPLC grade methanol added and each tube incubated at 40 °C for 15 min then filtered through 0.2 µm nylon membrane syringe filters (Chromatography Direct, Runcorn, Cheshire, UK) into HPLC vials and running through the chromatographic system. The HPLC ran using a H5C18-25QS (4.6×250 mm Interchrim, Montluçon Cedex, France) column with guard column [Phenomenex (Macclesfield, Cheshire, UK) KJ0-4282 SecurityGuard analytical guard cartridge system, fitted with an AJ0-7510 cartridge]. The effluent analysed comprised of 100 % HPLC grade methanol (Thermo Fisher Scientific, Loughborough, Leicestershire, UK) at a flowrate of 1 ml min^−1^ for 15 min, with an injection volume of 10 µl. Ultraviolet (UV) detection was set at a wavelength of 282 nm. Ergosterol produced a peak at a retention time of 8.1 min and standards were run at known concentrations (10 µg ml^−1^ to 200µg ml^−1^) to allow the construction of a standard curve for soil ergosterol quantification.

### Fungal biomass determination

Fungal biomass was determined from measured ergosterol concentration using equation 1 [32]:

Fungal biomas (FB)(μg/g)=Ergosterol(μg/g)×f×Rf

where *f* is 250 and *Rf* (recovery factor) is 1.61.

### Glomalin-related soil protein (GRSP) extraction

Glomalin-related soil protein (GRSP) was extracted via a modified methodology from Wright and Upadhyaya [33] to measure soil glomalin. Briefly, 1 g of soil was suspended in 8 ml 50 mM trisodium citrate dihydrate (Thermo Fisher Scientific, Loughborough, Leicestershire, UK) and kept at autoclave conditions (121 °C, 15 p.s.i.) for 60 min. Soils were then centrifuged at 1000 ***g*** for 2 min to remove suspended soil particles. Supernatant was further centrifuged at 6800 ***g*** for 10 min, a total of three times to remove impurities within the sample. Of the centrifuged sample, 1 ml was used for the Bradford protein assay (Coomassie Protein Assay Reagent, Thermo Fisher Scientific, Loughborough, Leicestershire, UK) at a photospectrometer (Cecil 1021, Cambridge, UK) absorbance of 595 nm.

### Determination of intracellular arbuscular structures

The staining and quantification process provided by Wilkes *et al*. [34] was utilised for 1 cm root sections of Zulu wheat via destructive sampling per week of plant growth. In brief, sampled root systems were submerged in excess acidified ethanol (50 % ethanol, 5 % acetic acid) for 24 h before triple rinsing in distilled water. Root samples were subsequently autoclaved at 121 °C, 15 p.s.i. for 15 min. Roots were cleared of debris via 10 min sonication in a 42 KHz ultrasonic water bath followed by further debris removal with a soft bristled paint brush. Roots were then transferred to 5 % hydrochloric acid and placed in a 60 °C water bath for 30 min. Roots were sectioned into 1 cm lengths in replicates of 5 per sample. Sectioned roots were placed in 10 % Sheaffer blue (10 % Sheaffer blue ink, 25 % acetic acid) for 3 min followed by a 1 min destaining step in distilled water. Stained roots were placed between a microscope slide and cover slip with light pressure applied to achieve a thin layer of cells for viewing. The counting of stained root vesicles and arbuscules was performed at a total magnification of 100×. Images of samples were taken with a Bresser HD microscope camera. Quantification of arbuscular density was chosen following the proportionality of arbuscular density to percentage colonisation [35, 36].

### Crop measurements

Crop height and root length were determined by utilizing a 1 m measuring tape place adjacent to the uprooted plant. Tiller count was achieved by visual counting. Root dry mass was quantified by the physical removal of adhered soil debris with a soft bristled paint brush, initial mass was measured before placing root systems (removed from the rest of the plant by cutting at the root collar) in a drying oven at 60 °C for 24 h. Root mass was weighed again for dry mass.

### Molecular analysis of fungal genera

Soil DNA was extracted via GeneAll Exgene soil (Cambio, Cambridge, UK), following the manufacturer’s instructions, and quantified via NanoDrop One/One microvolume UV-Vis spectrometer (Thermofisher, MA, USA). DNA samples were sent to Eurofins Genomics (Ebersberg, Germany) for ITS1 (fwd: GGAAGTAAAAGTCGTAACAAGG and rev: GCTGCGTTCTTCATCGATGC) and ITS2 (fwd: GCATCGATGAAGAACGCAGC and rev: TCCTCCGCTTATTGATATGC) illumina sequencing. Sample reports were produced by Eurofins Genomics and used to indicate AM fungal relative abundance. Fungal diversity was analysed via *k*-mer analysis protocols proved by [37].

### Statistics

Statistical analyses were conducted using R (version 4.1.0) and the extension package R commander (Hamilton, ON, Canada). The mean and standard error were calculated for each set of sample data. All quantified attributes were subjected to a Kolmogorov–Smirnov normality test. A multi-variate ANOVA tested for differences between inoculum treatments, tillage regime and type of inoculum at the time of sampling and at the end of the study. A single-factor ANOVA tested for differences between inoculum treatments within the same tillage regime. Where significant differences were identified, equal variance paired *T* tests of were employed for post hoc testing within the same tillage type or soil amendment, followed by Bonferroni corrections. Further paired two-tail *T* tests of unequal variance were applied to sample analysis between tillage treatments (the soil disturbance was not equal). Statistical significance was determined by *P* values≤0.05. Quantified crop and fungal attributes were subject to Pearson’s correlations. Linear regression analysis was also performed between all quantified crop growth and fungal attributes.

## Results

The compost amendment was determined to contain 13.27 mg l^−1^ total nitrogen, 3ppm phosphate (PO_4_), 3ppm potassium, and 28 % carbon from loss on ignition. NPK values of sampled soils, prior to soil amendment or inoculation, were determined to be that of Table 1.

**Table 1.** Determined total nitrogen (N), phosphate (PO_4_) (P), and potassium (K) of field sampled soils for their respective tillage and soil type

Tillage	Soil type	Nitrogen (mg l^−1^)	Phosphate (ppm)	Potassium (ppm)
Reduced	Sandy loam	10.19	2	2
	Clay loam	9.95	2	2
Conventional	Sandy loam	10.27	2	2
Zero	Sandy loam	11.89	2	2
	Sand	10.63	2	2
	clay	9.79	2	2

### Wheat and mycorrhizal biomass

A Kolmogorov–Smirnov normality test for all quantified attributes was seen to be *P*=0.24. A multi-variate ANOVA was able to show significance (*P*<0.0001) between tillage type, soil type, compost soil amendment and mycorrhizal inoucla, as well as each attribute individually. Further analysis of linear regression between all fungal and crop attributes of each tillage and soil type presented with a high degree of significance (*P*<0.00001). Regression statistics were noted to be the same between all tillage and soil types of each control (non-amended), compost amended and mycorrhizal inoculated soils. Statistical breakdown is provided in Tables S1, S2 and S3, available in the online version of this article, for control, compost and mycorrhizal inoculated soils, respectively.

**Fig. 1.**
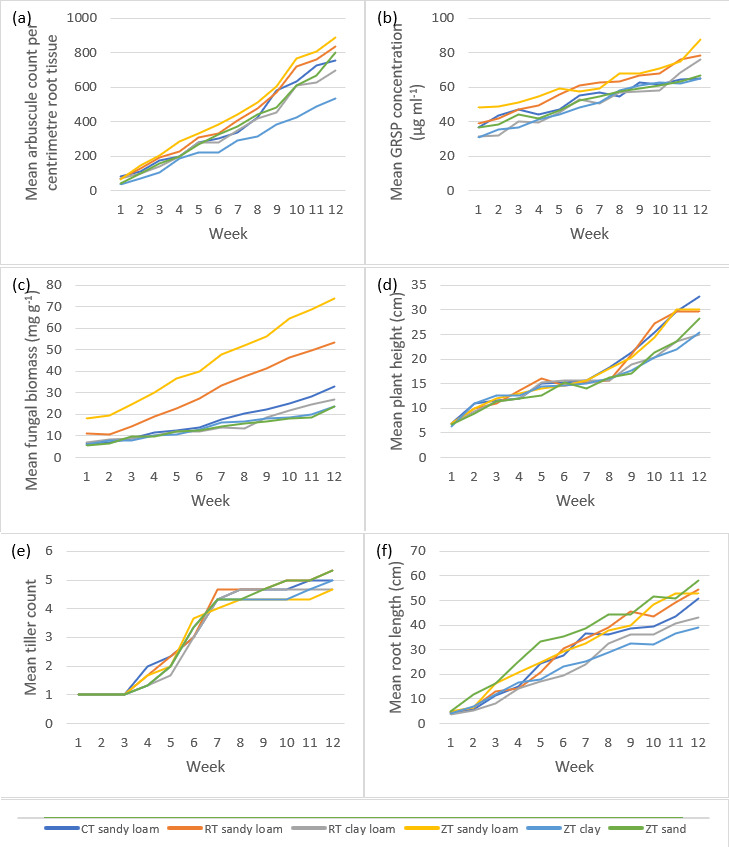
Control (non-soil amendment) soils supporting Zulu variety winter wheat (*n*=216 overall) over 12 weeks under controlled conditions for three tillage types (conventional tillage – CT, reduced tillage – RT and zero tillage – ZT) and four soil types (sandy loam, clay loam, clay and sand). Arbuscular mycorrhizal (AM) fungi were quantified by characteristics of growth, abundance and host symbiosis (**a–c**), including glomalin-related soil protein (GRSP), whilst crop growth and development were quantified by biomass parameters (**d–f**).

**Fig. 2.**
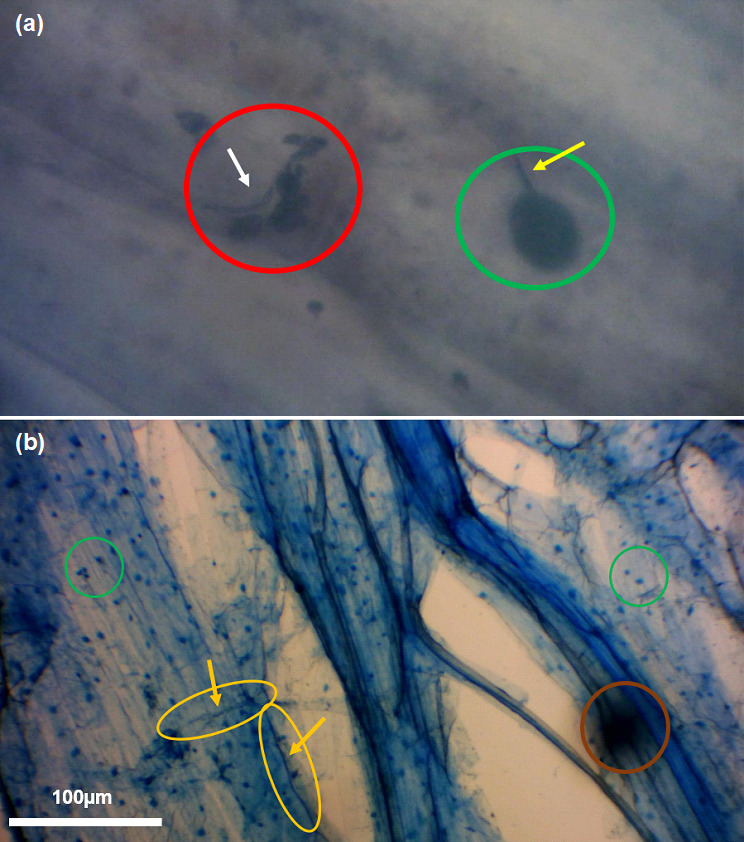
Stained root structures of winter wheat from sandy loam soils at a magnification of x1000 (**a**) and x400 (**b**) using an Apex microscope and imaged with a Bresser HD microscope camera. Red circle: developing juvenile arbuscule. Green circle: vesicle. Yellow circle and arrow: intraradical hyphae. Brown circle: debris. White arrow: peri arbuscular membrane.

Over a 12 week growth period, Zulu variety winter wheat tiller numbers, plant height and root length were seen to have been influenced by soil amendments (*P*<0.0001, degrees of freedom (df): 2, 645, F value: 456.61, F critical: 3.01, single factor ANOVA), as well as quantified AM fungal root arbuscules, glomalin and fungal biomass (*P*<0.0001, df: 2, 645, F value: 194.65, F critical: 3.01, single factor ANOVA) (Fig. 1). Post hoc *T* testing was able to show commercial mycorrhizal inoculants had a profound negative effect every sampling week throughout the study (*P*<0.0001, df: 215, t.stat: 19.24, equal variance *T* test), whereas compost soil amendments were seen to have a significantly positive impact on both AM fungal symbiotic characteristics [root arbuscules (Fig. 2), glomalin and fungal biomass] as well as plant growth measurements (plant height, number of tillers and root length) (*P*<0.0001, df: 215, t.stat: −39.06, equal variance *T* test). Bonferroni corrections produced a TRUE result for all quantified attributes, both AM fungal and plant biomass, for all tillage and soil types across all sampling weeks. Tillage and soil type were noted to have a reduced degree of influence (*P*=0.02, df: 5, 210, F value: 2.66, F critical: 2.26, multi variate ANOVA) on both plant growth characteristics and AM fungal symbiotic attributes compared to compost and mycorrhizal inoculant soil amendments.

**Fig. 3.**
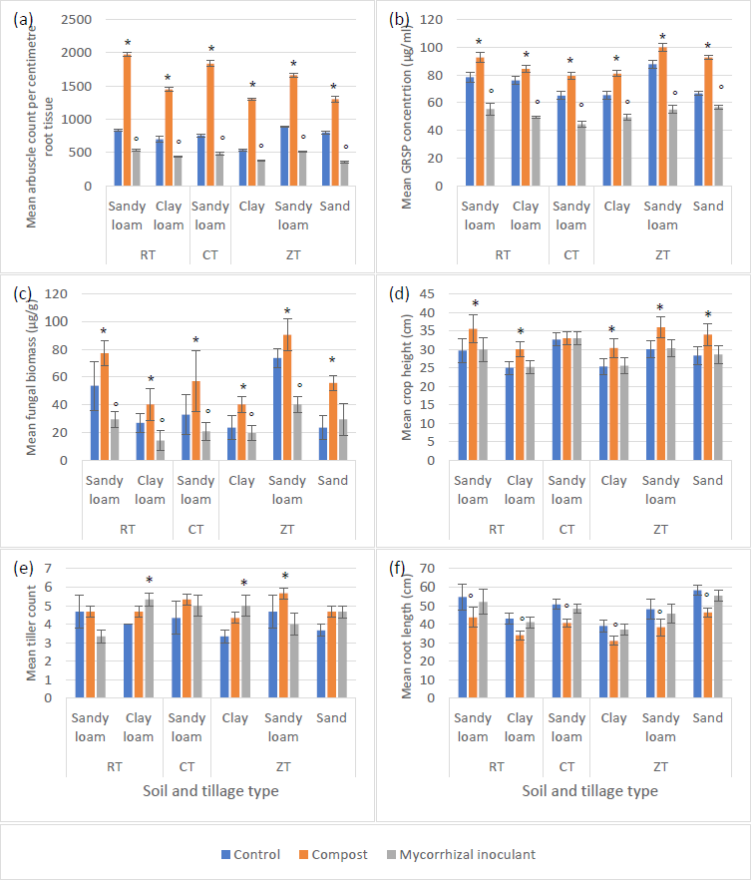
Arbuscular mycorrhizal (AM) fungal mean growth characteristic measurements, including glomalin-related soil protein (GRSP), (**a–c**) and mean crop growth measurements (**d–f**) for Zulu variety wheat plants at week 12 for non-treated control samples (*n*=18 per tillage per soil type), compost amended soil (*n*=18 per tillage per soil type) and mycorrhizal inoculated soil (*n*=18 per tillage per soil type) across three tillage types (CT – conventional tillage, RT – reduced tillage, ZT – zero tillage) and four soil types (sandy loam, clay loam, clay and sand). (*) indicates greatest significant increase (*P*<0.001), whereas (°) indicates greatest degree of significant decrease from the control (*P*<0.001). Error bars constructed from sem.

Compost-amended soils increased quantified AM fungal growth characterized by arbuscule counts per centimetre of root tissue, glomalin concentration and fungal biomass across all tillage and soil types at week 12 compared to control samples (*P*<0.0001, df:19,647, F value: 18.72, F critical: 2.12, multi-variate ANOVA). Mycorrhizal inoculant reduced AM fungal characteristics for each quantified attribute (Fig. 3a–c) for each tillage and soil type with the exception of ZT sandy soils showing a marginal increase (*P*=0.18). Crop height in CT sandy loam soil (d) did not benefit from any soil amendment. Root length was seen to reduce with the amendment of compost (*P*=0.002), marginal reduction was additionally seen from mycorrhizal inoculants (*P*=0.51).

Across the 12 week study, AM fungal characteristic, i.e. arbuscules, GRSP and fungal biomass, were seen to be significantly affected by soil texture (*P*<0.0001, df: 3, 212, F value: 23.35, F critical: 2.65, single factor ANOVA). Sandy soils were post hoc tested to and noted to have had the greatest negative influence on all AM fungal quantification (*P*<0.0001, df: 64, t.stat: 1.08, paired unequal variance *T* test). Crop biomass attributes, i.e. crop height, number of tillers and root length, produced a marginal significance between soil textures (*P*=0.02, df: 3, 212, F value: 4.96, F critical: 2.64, single factor ANOVA), with clay loam soils having the greatest reduction to crop biomass (*P*=0.02, df: 69, t.stat: 2.14, paired unequal variance post hoc *T* test).

Crop biomass and AM fungi were not quantified to be significantly different between tillage types throughout the 12 week growth of wheat (*P*=0.10, df: 2, 213, F value: 2.28, F critical: 3.03, single-factor ANOVA).

**Fig. 4.**
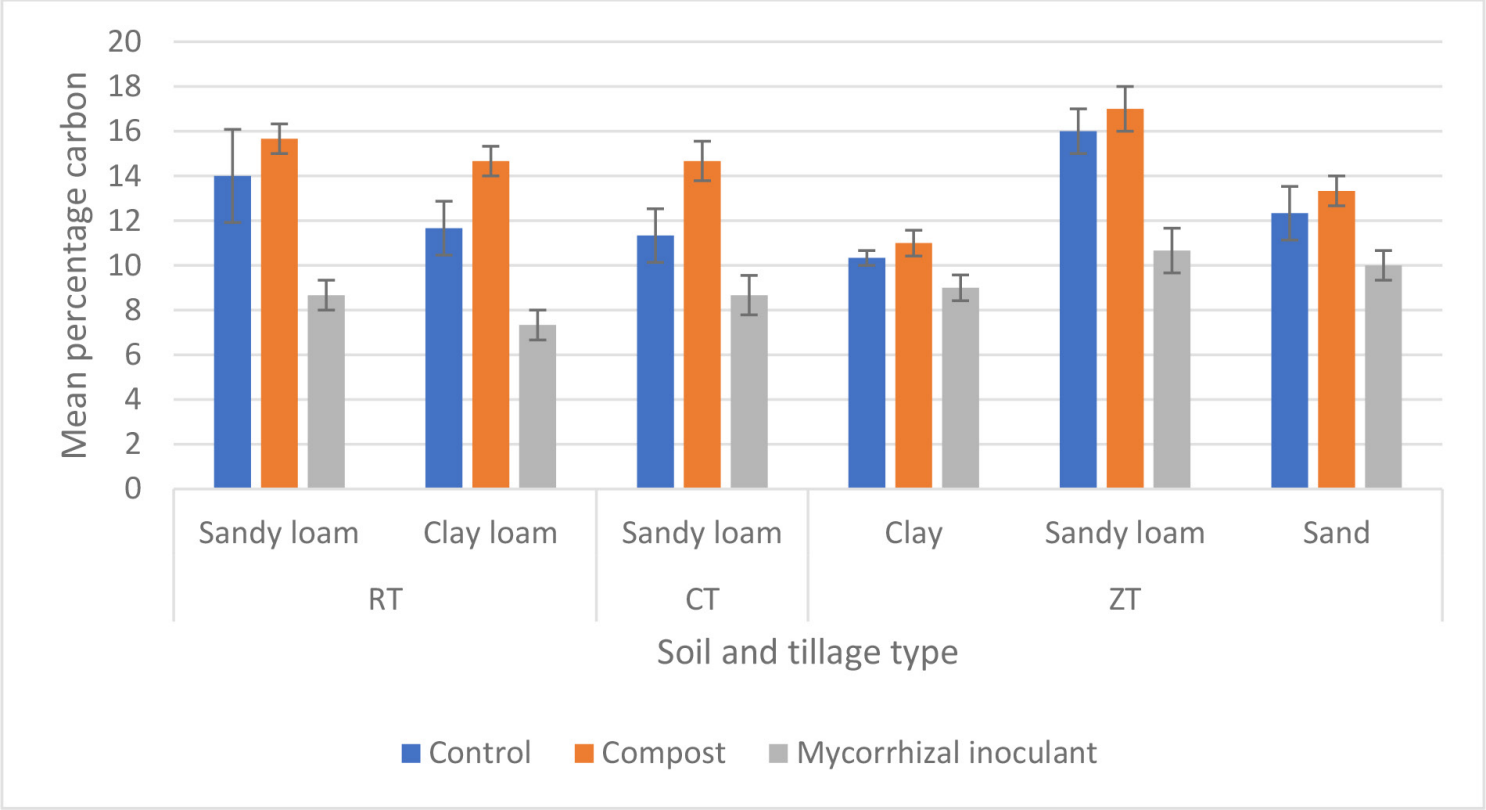
Mean (*n*=54 overall) percentage soil carbon, via loss on ignition, between four soil types and three tillage types (RT, reduced tillage; CT, conventional tillage; ZT, zero tillage) at week 12 of Zulu wheat growth. (*) indicates greatest degree of significant increase. Error bars constructed from sem.

Amending arable soils was seen to have a significant impact on soil carbon (*P*<0.001, df: 2,51, F value: 26.71, F critical: 3.18, single-factor ANOVA) (Fig. 4). Compost amended soils were quantified to contain a greater percentage of carbon, however, this was not seen to be a significant increase when compared to non-amended control soils from a post hoc *T* test (*P*=0.25, df: 16, t.stat: −0.66, paired equal variance *T* test). The same degree of significance was also observed between soil types of the same tillage between non-amended and compost-amended soils from further post hoc *T* testing. Mycorrhizal inoculant, however, was seen to significantly reduce quantified soil cardon at week 12 (*P*<0.001, df: 25, t.stat: 4.44, paired equal variance *T* test). Soil carbon percentage and GRSP were positively correlated between all soil and tillage types (0.65 Pearson’s correlation).

### Attribute correlations

Correlations for each attribute of control, compost and mycorrhizal inoculated soils are provided in the Supplementary Material (Figs S1, S2 and S3, respectively). Pearson’s correlations between all measured fungal and crop parameters are given in Tables S4, S5 and S6 for control, compost and mycorrhizal inoculated soils, respectively. Multi-variate ANOVA for all fungal and crop attributes between soil and tillage types showed significance (*P*<0.001).

**Fig. 5.**
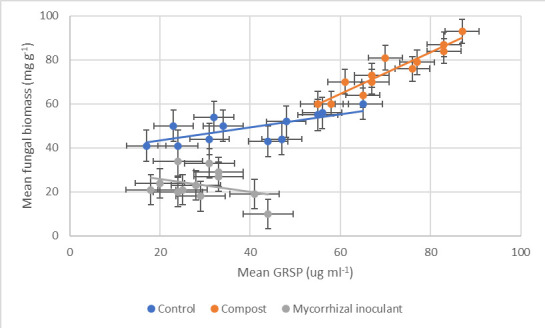
Correlation between mean glomalin related soil protein (GRSP) (*n*=216) and mean fungal biomass (*n*=216) for all tillage and soil types between the three treatments: control (no treatment) (Pearson’s correlation: 0.69), compost (Pearson’s correlation: 0.94) and commercial mycorrhizal inoculant (Pearson’s correlation: −0.35), at week 12. Error bars constructed from sem.

A strong correlation was observed between soil glomalin and fungal biomass in all soil and tillage types from soils amended with compost (Pearson’s correlation: 0.94) (Fig. 5). Both compost amendments and commercial mycorrhizal inoculant were seen to have a significant impact on the quantity of measurable glomalin (*P*<0.0001, df: 2, 42, F value: 42.46, F critical: 3.28, single-factor ANOVA) and fungal biomass (*P*<0.0001, df: 2, 42, F value: 120.32, F critical: 3.28, single-factor ANOVA) within sampled soils. Post hoc *T* tests further revealed mycorrhizal inoculant did not have a largely negative impact on soil glomalin (*P*=0.06, df: 215, t.stat: 19.24, paired equal variance *T* test), however, did have a profoundly negative influence on fungal biomass (*P*<0.0001). Compost-amended soils, on the other hand, were seen to produce a significantly greater quantity of soil glomalin (*P*<0.0001) and fungal biomass (*P*<0.0001) at week 12.

**Fig. 6.**
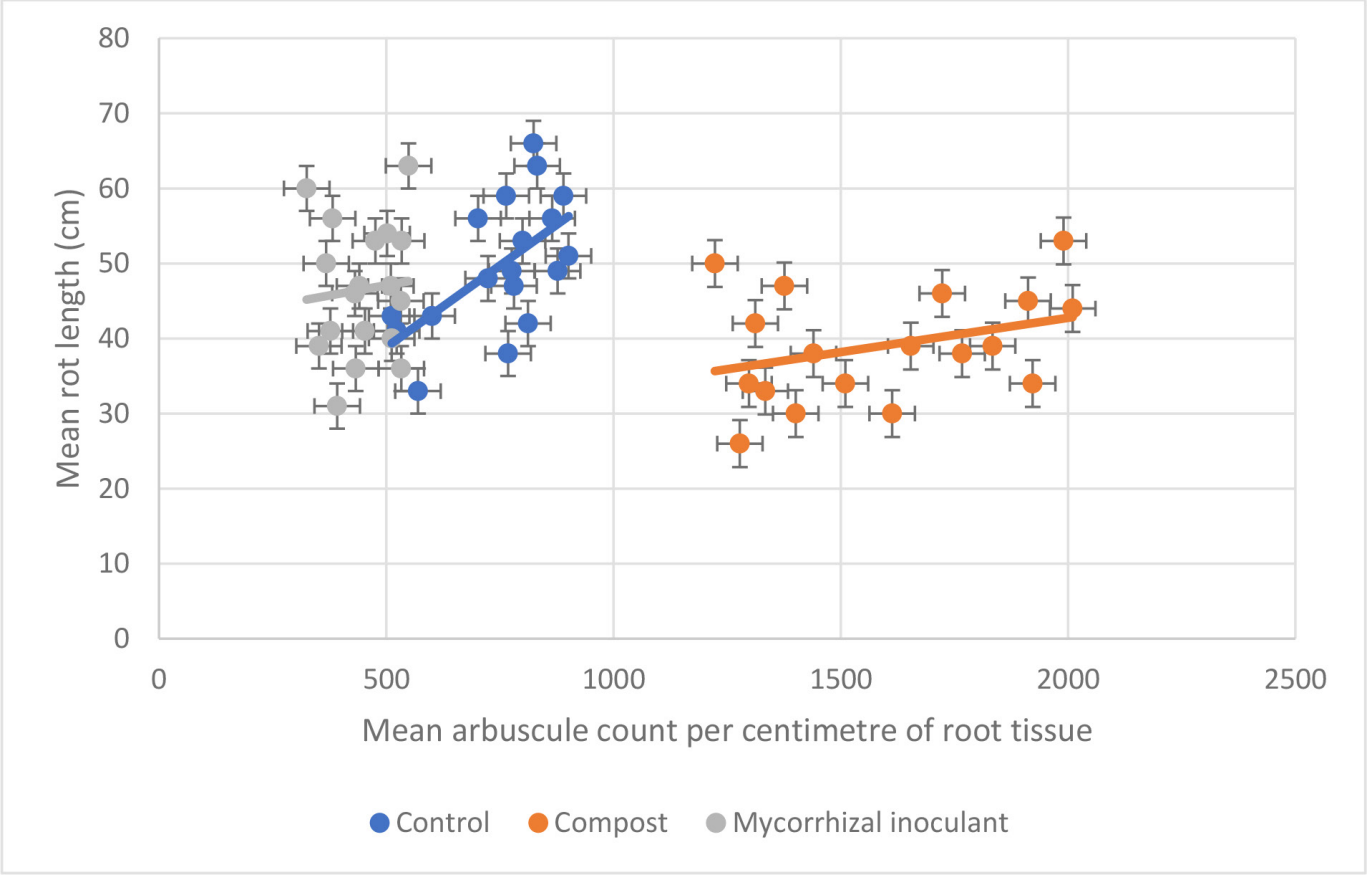
Correlation between mean root length (*n*=216) and mean arbuscle count (*n*=216) for all tillage and soil types between the three treatments: control (no treatment) (Pearson’s correlation: 0.47), compost (Pearson’s correlation: 0.33) and commercial mycorrhizal inoculant (Pearson’s correlation: 0.09), at week 12. Error bars constructed from sem.

Mycorrhizal inoculant was quantified to have reduced AM fungal root arbuscules in comparison to control non-treated soils (*P*<0.0001, df: 215, t.stat: 3.55, paired unequal variance *T* test), however, root length was not influenced to the same degree by the presence of the mycorrhizal inoculant (*P*=0.25) (Fig. 5). Compost-amended soils increased the density, and therefore quantity, of AM fungal root arbuscules (*P*<0.0001, df: 215, t.stat: −11.99, paired unequal variance *T* test) and reduced root length (*P*=0.01) compared to control plants (Fig. 6).

### ITS sequencing

**Fig. 7.**
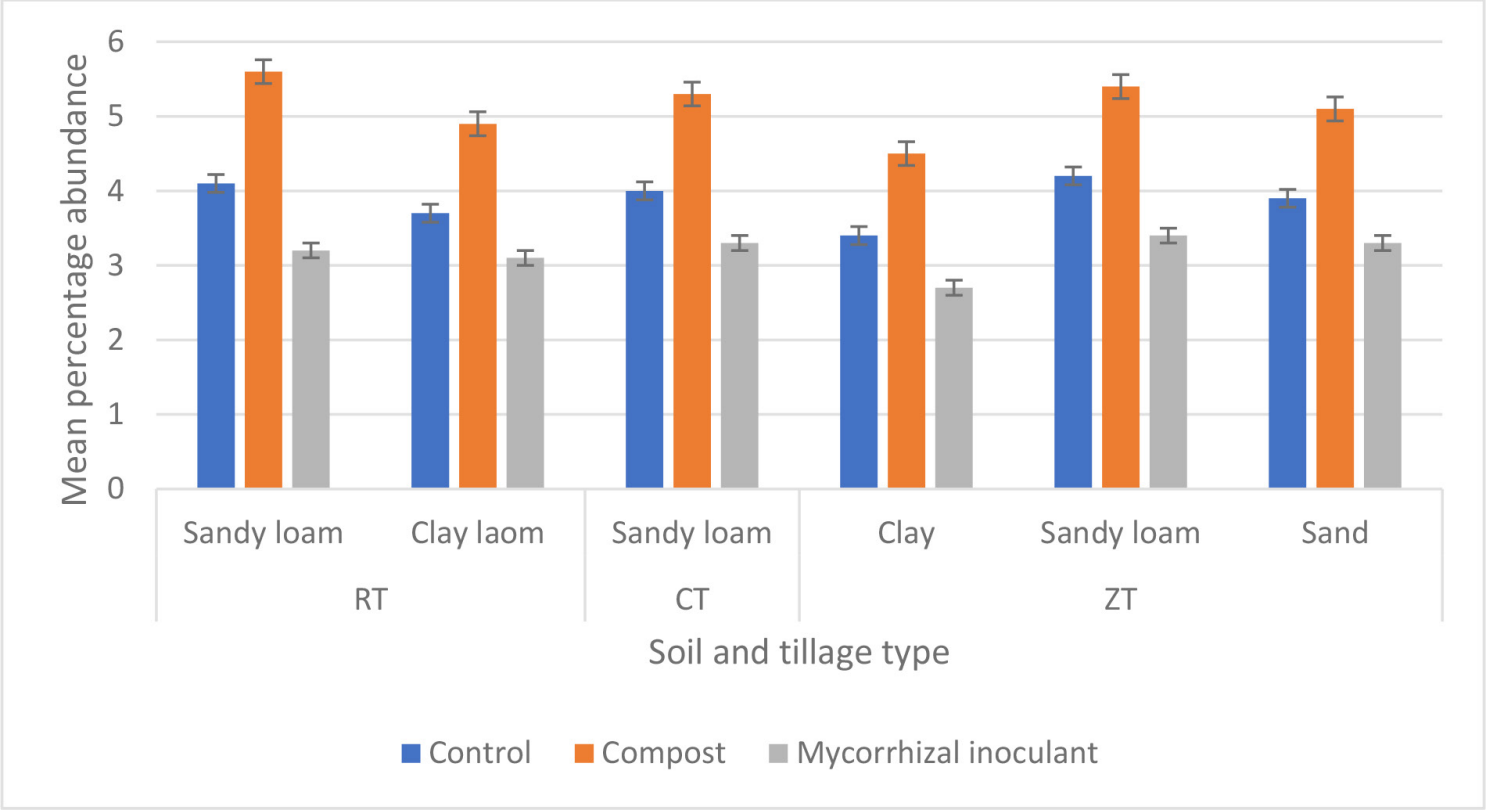
Mean percentage relative abundance of ITS1 and ITS2 sequences identified as arbuscular mycorrhizal (AM) fungi genera, from *k*-mer sequencing depth, across four soil types and three tillage practices after week 12 of wheat growth. RT, reduced tillage; CT, conventional tillage; ZT, zero tillage. Error bars constructed from sem.

Molecular ITS1 and ITS2 sequencing, focusing on AM fungal genera, (Fig. 7) indicated a significant increase in AM fungal relative abundance from the addition of compost (*P*<0.0001, df: 2,15, F value: 39.7, F critical: 3.68, single-factor ANOVA). Illumina sequencing identified a total of 59 genera across all soil samples, of these, there were 13 unique genera. Compost amended soils were additionally seen to have significantly increased the number of identified species (*P*<0.0001, df: 10, t.stat: −6.32, *T* test of equal variance), whereas a mycorrhizal inoculant did not have a significant influence on the number of identified AM fungal species (*P*=0.30, df: 10, t.stat: 0.54, *T* test of equal variance). Sequencing analysis indicated a total of 251 fungal species across all soil types, however, a gamma diversity of 24 fungal species was observed. Furthermore, sequencing analysis of the compost used for soil amendments indicated three species of mycorrhizal fungi present (*Rhizophagus intraradices*, *R. irrgularis* and *Funneliformis masseae*) (Table 2).

**Table 2.** Identified arbuscular mycorrhizal (AM) fungal species per genera from ITS1 and ITS2 sequencing after 12 weeks of wheat growth. RT, reduced tillage; CT, conventional tillage; ZT, zero tillage

Tillage type	Soil texture	Control			Compost			Mycorrhizal inoculant		
		*Glomus*	*Rhizophagus*	*Funneliformis*	*Glomus*	*Rhizophagus*	*Funneliformis*	*Glomus*	*Rhizophagus*	*Funneliformis*
RT	Sandy loam	Not detected	*intraradices irregularis*	*mosseae*	Not detected	*intraradices irregularis*	*mosseae*	aggregatum	*intraradices*	Not detected
	Clay loam	Not detected	*intraradices*	*mosseae*	Not detected	*intraradices irregularis*	*mosseae*	Not detected	*irregularis*	Not detected
CT	Sandy loam	Not detected	*irregularis*	Not detected	Not detected	*intraradices irregularis*	*mosseae*	*mosseae*	Not detected	Not detected
ZT	Clay	Not detected	*intraradices*	Not detected	Not detected	*intraradices irregularis*	*mosseae*	Not detected	*irregularis*	Not detected
	Sandy loam	*aggregatum*	*intraradices*	Not detected	Not detected	*intraradices irregularis*	*mosseae*	Not detected	*intraradices*	Not detected
	Sand	Not detected	Not detected	*mosseae*	Not detected	*intraradices irregularis*	*mosseae*	*geosporum*	Not detected	Not detected

Multi-variate ANOVA between tillage and soil type for both *Glomus* spp. and *Rhizophagus* spp., indicated a significant increase in AM fungal diversity from compost amended soils (*P*<0.001). A single factor ANOVA further indicated a significant increase in both AM fungal genera from the amendment of compost into arable soils (*P*<0.0001, df: 5,30, F value: 13.76, F critical: 2.53), with RT sandy loam soils receiving the greatest increase in relative abundance of AM fungi (*P*<0.0001. df: 10, t.stat: 0.62, equal variance post hoc *T* test). Shannon indicies are shown in Table 3, with beta diversities shown in Tables 4–6.

**Table 3.** Shannon indices of identified fungal sequences for all soil types across three tillage types: RT (reduced tillage), CT (conventional tillage) and ZT (zero tillage)

Tillage type	Soil type	Control soils	Compost-amended soils	Mycorrhizal inoculated soils
RT	Sandy loam	3.26	2.90	0.68
	Clay loam	1.95	2.62	1.48
CT	Sandy loam	1.70	1.82	1.13
ZT	Clay	1.09	1.57	0.38
	Sandy loam	1.13	2.53	0.80
	Sand	0.39	1.94	1.51

**Table 4.** Beta diversity of all identified fungal sequences in control (non-amended soils) between their respective soil and tillage types. RT (reduced tillage), CT (conventional tillage) and ZT (zero tillage)

Tillage type		RT		CT	ZT		
	Soil type	Sandy loam	Clay loam	Sandy loam	Clay	Sandy loam	Sand
RT	Sandy loam						
	Clay loam	20					
CT	Sandy loam	18	9				
ZT	Clay	18	8	2			
	Sandy loam	14	10	6	6		
	Sand	15	13	5	7	7	

**Table 5.** Beta diversity of all identified fungal sequences between control (non-amended soils) and compost-amended soils of their respective soil and tillage types. (*) indicates a reduction to fungal diversity from the amendment of soil with compost. RT (reduced tillage), CT (conventional tillage) and ZT (zero tillage)

			Control					
	Tillage type		RT		CT	ZT		
		Soil type	Sandy loam	Clay loam	Sandy loam	Clay	Sandy loam	Sand
Compost	RT	Sandy loam	2					
		Clay loam		3				
	CT	Sandy loam			3			
	ZT	Clay				4*		
		Sandy loam					2	
		Sand						0

**Table 6.** Beta diversity of all identified fungal sequences between control (non-amended soils) and mycorrhizal inoculated soils of their respective soil and tillage types. (*) indicates a reduction to fungal diversity from the amendment of soil with a mycorrhizal inoculum. RT (reduced tillage), CT (conventional tillage), and ZT (zero tillage)

			Control					
	Tillage type		RT		CT	ZT		
		Soil type	Sandy loam	Clay loam	Sandy loam	Clay	Sandy loam	Sand
Mycorrhizal inoculant	RT	Sandy loam	11*					
		Clay loam		7*				
	CT	Sandy loam			2			
	ZT	Clay				1*		
		Sandy loam					3*	
		Sand						3

## Discussion

The present study has been able to show the effects of a commercial mycorrhizal inoculant and compost amended arable soils on wheat growth and AM fungal host associations. Compost amendments were able to increase AM fungal associations with a host throughout the 12 week study, whereas a commercial inoculant was seen to have reduced all AM fungal symbiotic characteristics whilst having minimal effects on crop growth and development.

Several studies have investigated the amendment of arable soils with a range of materials, including earthworms, compost, or biochar, prior to seed drilling/cultivation in attempts to increase the overall yield of the crop as well as increase soil quality [28–31, 38]. Growing cowpeas (*Vigna unguiculata*), common beans (*Phaseolus vulgaris*) and wheat (*Triticum aestivum L*.), Cobb *et al*. [39] was able to identify increases in AM fungal interactions with a host plant in compost and biochar amended soils compared to non-amended soils. The present study, whilst not growing wheat in silty soils, is able to show reductions in AM fungal abundance and their interactions with host wheat in clay and clay loam soils compared with other soil textures. This has also been shown by Vieira *et al*. [40]. A potential explanation to the reduction in AM fungal abundance and host interaction noticed by Cobb *et al*. [39] may come, in part, from the soil texture employed utilising findings of the present study. Ndiate *et al*. [41] further amended soils with biochar, but did not employ compost as a soil amendment, for increased soil sustainability, wheat yield, and AM fungal–host interactions, along with *Funneliformis mosseae* colonized root as a soil inoculant produced from the controlled growth of maize (*Zea mays*) for 4 months. The present study used a commercially available mix of mycorrhizal fungi and noticed reductions in AM fungal–host interactions and no significant influence on crop growth. With the single species inocula by Ndiate *et al*. [41], root dry mass was seen to have increased compared to non-inoculated soils. As further shown in the present study, and by Wilkes *et al*. [17] and Wilkes [30], root length is typically reduced with the increase in AM fungal–host root interaction, making these attributes negatively correlated. Therefore, a reduction in root mass would have been expected by Ndiate *et al*. [41]. As also demonstrated by Wilkes *et al*. [17], increases in root length and mass following AM fungal inocula is typically resultant of antagonistic bacterial–mycorrhizal interactions, resulting in reduced AM fungal biomass and subsequent reductions in the mycorrhizal–host relationship. Furthermore, stained root sections by Ndiate *et al*. [41] were seen to be greatest in soils amended with biochar compared with single AM fungal species inoculum. However, as shown by Wilkes *et al*. [36], trypan blue root staining, utilised by Ndiate *et al*. [41], does not allow for sufficient arbuscular quantification. As also described by Wilkes [31], employment of potassium hydroxide during root staining has the potential to damage root cortical arbuscules, further reducing their quantification. Increases in quantified root arbuscules and fungal biomass in compost-amended soils of the present study were also reported by Yang *et al*. [42]. Molecular analysis from Yang *et al*. [42] focused upon AM fungal population diversity and were also able to suggest that the bacterial community within compost aids in the further growth and development of AM fungi and their extra radiating hyphae into bulk soils. An example of this can be seen from mycorrhizal helper bacteria (MHB) [43]. Whilst bacterial investigations were not performed in the present study and not the focus of molecular analysis by Yang *et al*. [42], further investigation is warranted to determine MHB populations and contributions to AM fungal development from compost amended soils.

Several studies have been able to show the reduction in AM fungal abundance in soil managed by either CT or ZT, with a recovery of AM fungal abundance and the degree of host interactions, via quantified root arbuscles, in CT-managed soils [8, 44–46]. However, the implementation of glyphosate in ZT was also reported to have detrimental influences on AM fungal abundance and host interactions [17] with a slower rate of AM fungal abundance recovery. Arbuscular mycorrhizal fungi were consistently more abundant in ZT soils, regardless of glyphosate applications, when compared with CT soils. Reductions to AM fungal population and their abundances has been reported by several studies to be correlated with increases in soil loss via erosion, which is also correlated to reduced quantities of adhesive glomalin stabilizing soil aggregates and reducing soil erosion [11, 45–51]. The present study built upon results demonstrated by previous investigations [17, 30] in order to suggest potential practical management strategies to increase AM fungal abundance and their associated interactions with a host crop. This would have benefits for both soil quality as well as plant health, growth and development. From data presented in (Fig. 3) at week 12, AM fungal–host interaction and abundance attributes were seen to be significantly increased (*P*<0.0001) compared to control non-amended soils for all tillage and soil types investigated. Furthermore, crop height and the number of tillers per plant were seen to be greater in soils amended with compost compared to control soils and soils provided with a commercial mycorrhizal inoculum. This shows the benefit to crop development from the presence of a well-developed and abundant AM fungal population with soils supporting such a crop. Fig. 3f, however, does show reduced crop root length in soils amended with compost. This is not a negative effect, rather an advantageous benefit to the crop. As shown by Wilkes *et al*. [17] and Wilkes *et al*. [30], by increasing an AM fungal population, and subsequent host interactions by the increased density of root cortical arbuscules, the mycorrhizal hyphal network adopts some of the functionality of the host’s root system. This allows the host crop to utilise acquired soil nutrients and photosynthate for above ground growth and development, as suggested by Fig. 3d and e. Sandy soils under ZT management were observed to have the greatest increase in GRSP and fungal biomass at week 12 compared to other soil and tillage types. Wilkes *et al*. [30] also reported the reduced abundance of AM fungi in sandy soils under ZT management compared with other soil types under the same tillage. The present study has been able to produce evidence that sandy soils, under ZT, benefit from the addition of compost to support AM fungi, as well as produce an AM fungal population to support crop growth and development. Other soil types under ZT management, whilst increases were noted in AM fungal attributes, did not increase to the same degree as sandy soils amended with compost. Above ground plant growth characteristics were also seen to be generally greater with the addition of compost as opposed to the commercial mycorrhizal inoculant. Crop height was increased for RT and ZT, along with their respective soil types. CT-managed soils were the exception where crop height was not seen to have significantly increased between control soils and amended soils.

Increases in soil carbon have been shown to aid in the increased abundance of AM fungal populations [51–53], as well as having benefits to increasing soil quality [54, 55]. It is to be expected that amending soils with compost will also provide additional soil carbon, however, as shown by (Fig. 4), compost amendments did not significantly provide a greater quantity of soil carbon in the quantities amended into sampled arable soils. A mycorrhizal inoculant, on the other hand, was seen to have significantly reduced the quantity of soil carbon after 12 weeks of wheat growth. Fungal biomass and GRSP, contributors to soil carbon, were also both reduced in soils inoculated with a commercial mycorrhizal product. This may suggest an antagonistic competition between the mycorrhizal species with the commercial product, as previously shown between mycorrhizal species by several studies [56–60], under the conditions employed in the present study. This may provide a further explanation for the reduced quantification of AM fungal attributes seen from soils that received a commercial mycorrhizal inoculum. Furthermore, competition of mycorrhizal species may also be an explanation for no change in crop height and root length observed from the commercial mycorrhizal inoculum.

From an overview of all data presented in the current study, the mycorrhizal inoculum has the potential to have caused multi-species competition with mycorrhizal populations already within arable soils, resulting in the reduced AM fungal characteristics of symbiosis recorded at week 12 (Figs 1a–c, 3a–c, 4 and 6), also described by Berruti *et al*. [22] and Yang *et al*. [49]. From information provided by the manufacturers, the commercially produced mycorrhizal spores are a combination of both AM fungi and ectomycorrhizal (EcM) fungi. The commercial inoculant of mycorrhizal spores, in the present study, was utilised with wheat, a known AM fungal host. However, following the reasoning provided by Berruti *et al*. [22] and Yang *et al*. [49], as well as data provided in the present study, the commercially available mycorrhizal inoculant had negative implications for AM fungal symbiosis as quantified by root arbuscules, GRSP, and fungal biomass via ergosterol. Molecular investigations of the present study (Fig. 7 and Table 2) show a reduction in mycorrhizal relative abundance and diversity (Table 3), while adding further evidence to the reasoning provided by Berruti *et al*. [22] and Yang *et al*. [49]. Control non-amended soils across all soil and tillage types in conservational tillage practices, i.e. ZT and RT, would suggest that mycorrhizal fungi are conserved under these practices, whilst CT-managed soils are detrimental to mycorrhizal diversity, further be seen from low beta diversities in (Table 4). This has also been shown by Kabir [8], Sheehy *et al*. [9], Wilkes *et al*. [17], Wilkes *et al*. [30], and Bendini *et al*. [50]. The addition of compost as a soil amendment was noted to have increased the overall abundance of mycorrhizal fungi. However, as shown by (Table 2), only three species of mycorrhiza were then detected from Illumina sequencing. This is likely due to the greater abundance these species within the compost, as well as species competition within the soils as alluded to by Berruti *et al*. [22] and Yang *et al*. [49]. Interestingly, the inoculation of soils with a commercial mycorrhizal inoculant reduced the overall abundance and diversity of identifiable mycorrhizal species. Engelmoer *et al*. [61] studied the interaction between *R. intraradices* and *G. aggregatum* in the root microbiome. Engelmoer *et al*. [61] were able to show the reduction of mycorrhizal abundance and root colonisation in the presence of the combination of mycorrhizal species. However, if mycorrhiza were present as a monoculture, root colonisation and mycorrhizal abundance were approximately three times greater. This can also be shown from the overall mycorrhizal abundance in (Fig. 7), identified species in (Table 2), and the reduction of root arbuscules (a marker of mycorrhizal root colonisation) from the commercial inoculant in (Fig. 3a).

Crop growth measurements were not seen to maintain the correlations seen with AM fungal abundance. Whilst most quantified crop growth parameters were noted to have reduced in soils amended with a commercial mycorrhizal source, the number of wheat tillers (Fig. 3e) were observed to have produced a greater increase from the addition of a commercial mycorrhizal inocula compared to compost amendments in clay soils under ZT and RT. This may suggest clay soils and their physical soil characteristics produce favourable conditions towards species within the commercial inocula. Further investigation is warranted to underpin the interactions between multi mycorrhizal species and soil type. The study by Wilkes *et al*. [30] used soils from the same sample sites as the present study and was able to provide details regarding mycorrhizal abundances within each soil type. Presented data by Wilkes *et al*. [30] was able to show AM fungal abundance greatly reduced in CT sandy loam, ZT clay, and ZT sandy soils; the same soil types that produced an increase in the number of wheat tillers from the mycorrhizal incoula compared to control non-amended soils, whilst not being significantly different from compost amendments. This may suggest that such an inoculum source of mycorrhizal fungi is better suited for mycorrhizal depleted or reduced soils as opposed to soils with a greater abundance of mycorrhiza such as ZT and RT sandy loam, indicating that a tailored approach to mycorrhizal soil amendments is dependent on soil type and requiring further investigation.

## Conclusions

The present study is able to conclude that arable soils amended with compost provide a greater abundance of AM fungi within soils for the sustained growth and development of winter wheat. Further study is required to substantiate this for other crop types, along with a greater range of soil types. Furthermore, based on the cost to purchase the commercial mycorrhizal inocula, large-scale application of compost is more cost effective. Additionally, the method in which the commercial inoculant is to be added to soils, as a layer underneath the root systems of the developing plant according to the manufacturer’s instructions, is not a practical method of application for field-scale crop development. The present study did not follow this recommendation after performing several samples under this advice and not recording a significant difference (*P*=0.87) between a layer of inocula compared to a homogenized inocula (data not shown). Method comparatives of compost incorporation in soils were able to show that no further benefit was achieved to crop development and AM fungal populations if compost was applied as a layer or homogenized into topsoil. The only requirement for soils to be amended with compost, was to apply the compost before cultivating or seed drilling.

## Supplementary Data

Supplementary material 1
