# Supplementary material for: The influence of a soil amendment on the abundance and interaction of arbuscular mycorrhizal fungi with arable soils and host winter wheat
Source: Access Microbiol. 2024 Jan 16;6(1):000581.v5. doi: 10.1099/acmi.0.000581.v5 (PMC10866040; doi:10.1099/acmi.0.000581.v5)
Supplement: Supplementary material 1 [file acmi-6-581.v5-s001.pdf]

Supplementary table 1 – Linear regression breakdown for control (non amended) soils showing a significance of P<0.00001. All soil (Sand, sandy loam, clay, and clay loam) and tillage types (conventional, reduced, and zero tillage) presented with the same degree of significance and statistical breakdown. T stats are presented along with R^2 values in brackets.

	Arbuscules count	Carbon (%)	Crop height (cm)	Fungal biomass (mg/g)	Glomalin (ug/g)	Root dry mass (g)	Root length (cm)	Tiller count
Arbuscle count								
Carbon (%)	-5.75 (0.59)							
Crop height (cm)	-8.63 (0.84)	11.79 (0.43)						
Fungal biomass (mg/g)	8.18 (0.52)	27.68 (0.47)	15.25 (1.00)					
Glomalin (ug/g)	-13.32 (0.74)	0.45 (0.56)	-8.55 (0.53)	-8.55 (0.53)				
Rot dry mass (g)	-0.73 (0.85)	19.69 (0.51)	1.85 (0.40)	1.85 (0.40)	34.26 (0.69)			
Root length (cm)	-0.73 (0.85)	19.69 (0.51)	1.85 (0.40)	1.85 (0.40)	34.26 (0.69)	10.71 (0.40)		
Tiller count	2.39 (0.64)	19.35 (0.32)	3.64 (0.26)	3.64 (0.26)	30.25 (0.52)	4.20 (0.69)	4.20 (0.69)	

Supplementary table 2 – Linear regression breakdown for compost amended soils showing a significance of P<0.00001. All soil (Sand, sandy loam, clay, and clay loam) and tillage types (conventional, reduced, and zero tillage) presented with the same degree of significance and statistical breakdown. T stats are presented along with R^2 values in brackets.

	Arbuscules count	Carbon (%)	Crop height (cm)	Fungal biomass (mg/g)	Glomalin (ug/g)	Root dry mass (g)	Root length (cm)	Tiller count
Arbuscle count								
Carbon (%)	-2.69 (0.58)							
Crop height (cm)	2.18 (0.82)	16.06 (0.59)						
Fungal biomass (mg/g)	9.61 (0.63)	22.03 (0.38)	10.36 (0.63)					
Glomalin (ug/g)	-6.70 (0.69)	3.29 (0.55)	-9.29 (0.79)	-8.98 (0.67)				
Rot dry mass (g)	11.96 (0.73)	27.41 (0.63)	14.23 (0.82)	6.02 (0.62)	43.16 (0.82)			
Root length (cm)	11.96 (0.73)	27.41 (0.63)	14.23 (0.82)	6.02 (0.62)	43.16 (0.82)	10.62 (1.00)		
Tiller count	7.86 (0.61)	20.15 (0.43)	8.43 (0.66)	5.19 (0.38)	25.23 (0.57)	0.91 (0.67)	0.91 (0.67)	

Supplementary table 3 – Linear regression breakdown for mycorrhizal inoculated soils showing a significance of P<0.00001. All soil (Sand, sandy loam, clay, and clay loam) and tillage types (conventional, reduced, and zero tillage) presented with the same degree of significance and statistical breakdown. T stats are presented along with R^2 values in brackets.

	Arbuscules count	Carbon (%)	Crop height (cm)	Fungal biomass (mg/g)	Glomalin (ug/g)	Root dry mass (g)	Root length (cm)	Tiller count
Arbuscle count								
Carbon (%)	-3.11 (0.46)							
Crop height (cm)	-5.40 (0.70)	10.0 (0.41)						
Fungal biomass (mg/g)	3.41 (0.59)	20.31 (0.56)	14.26 (0.47)					
Glomalin (ug/g)	-8.59 (0.59)	0.08 (0.53)	-1.67 (0.48)	-6.44 (0.51)				
Rot dry mass (g)	0.44 (0.77)	18.40 (0.50)	14.17 (0.72)	3.72 (0.58)	30.93 (0.55)			
Root length (cm)	0.44 (0.77)	18.40 (0.50)	14.17 (0.72)	3.72 (0.58)	30.93 (0.55)	-0.10 (1.00)		
Tiller count	1.09 (0.50)	14.73 (0.26)	9.59 (0.52)	4.16 (0.30)	23.12 (0.27)	2.14 (0.55)	2.14 (0.55)	

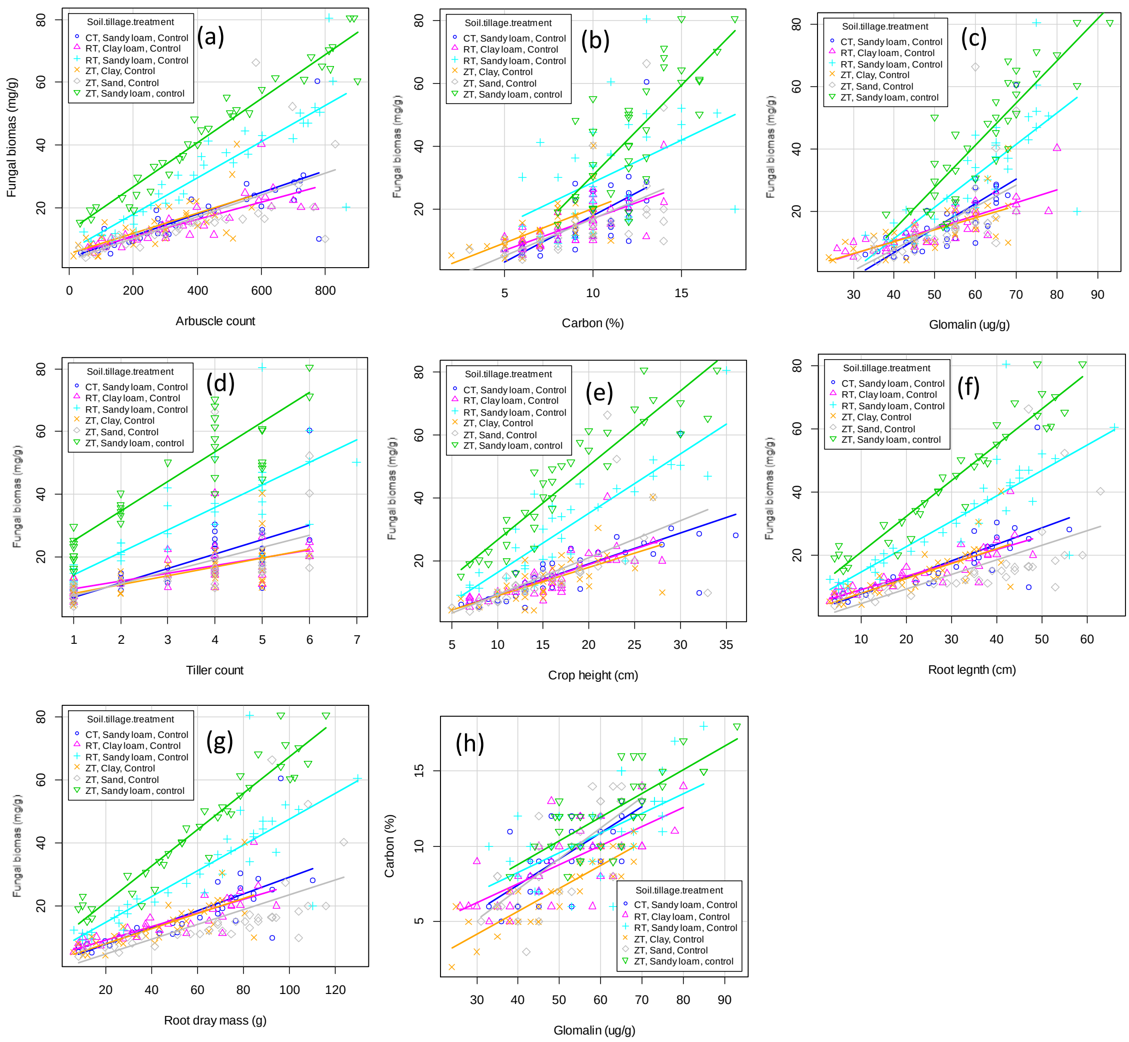

Supplementary figure 1 – Corrected relationships between fungal biomass and (a) arbuscule count, (b) percentage carbon, (c) glomalin, (d) tiller count, (e) crop height, (f) root length, as well as (g) root dry mass in control (non-amended) soils of 4 soil textures (sandy loam, clay loam, clay and sand), as well as between 3 tillage types (conventional, reduced, and zero). Correlation between soil glomalin and carbon between the mentioned soil textures and tillages can be seen in (h).

Supplementary table 4 – Presented R^2 values for Pearson's correlations of fungal and crop growth attributed shown in Supplementary figure 1.

	Arbuscules count	Carbon (%)	Crop height (cm)	Fungal biomass (mg/g)	Glomalin (ug/g)	Root dry mass (g)	Root length (cm)	Tiller count
Arbuscle count								
Carbon (%)	0.77							
Crop height (cm)	0.92	0.66						
Fungal biomass (mg/g)	0.72	0.69	0.63					
Glomalin (ug/g)	0.86	0.75	0.79	0.73				
Rot dry mass (g)	0.92	0.78	0.85	0.64	0.83			
Root length (cm)	0.92	0.78	0.85	0.64	0.83	1.00		
Tiller count	0.80	0.57	0.72	0.51	0.72	0.83	0.83	

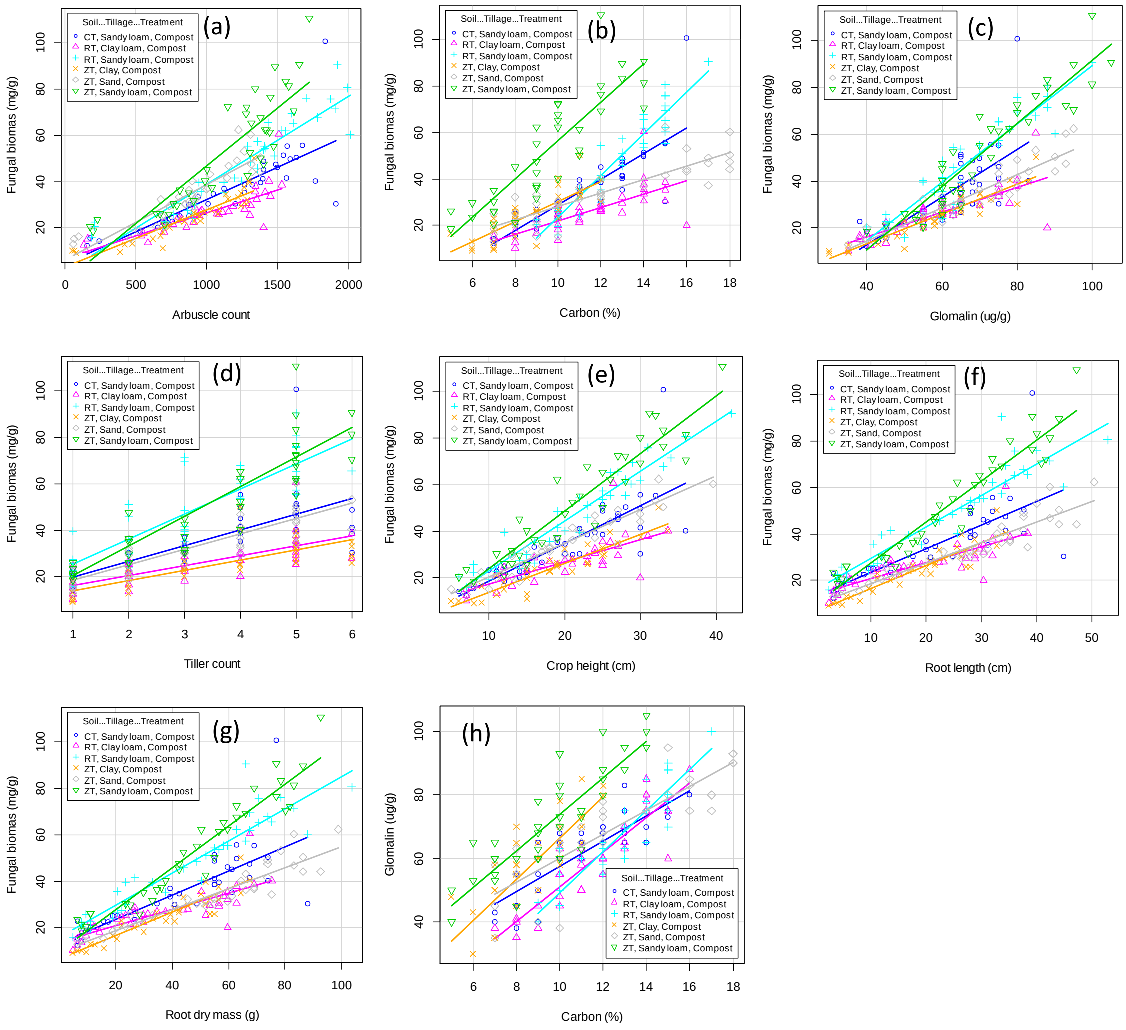

Supplementary figure 2 – Corrected relationships between fungal biomass and (a) arbuscule count, (b) percentage carbon, (c) glomalin, (d) tiller count, (e) crop height, (f) root length, as well as (g) root dry mass in compost soils of 4 soil textures (sandy loam, clay loam, clay and sand), as well as between 3 tillage types (conventional, reduced, and zero). Correlation between soil glomalin and carbon between the mentioned soil textures and tillages can be seen in (h).

Supplementary table 5 – Presented R^2 values for Pearson's correlations of fungal and crop growth attributed shown in Supplementary figure 2.

	Arbuscules count	Carbon (%)	Crop height (cm)	Fungal biomass (mg/g)	Glomalin (ug/g)	Root dry mass (g)	Root length (cm)	Tiller count
Arbuscle count								
Carbon (%)	0.76							
Crop height (cm)	0.90	0.77						
Fungal biomass (mg/g)	0.79	0.62	0.80					
Glomalin (ug/g)	0.83	0.74	0.89	0.82				
Rot dry mass (g)	0.85	0.79	0.91	0.79	0.91			
Root length (cm)	0.85	0.79	0.91	0.79	0.91	1.00		
Tiller count	0.78	0.66	0.81	0.62	0.75	0.82	0.82	

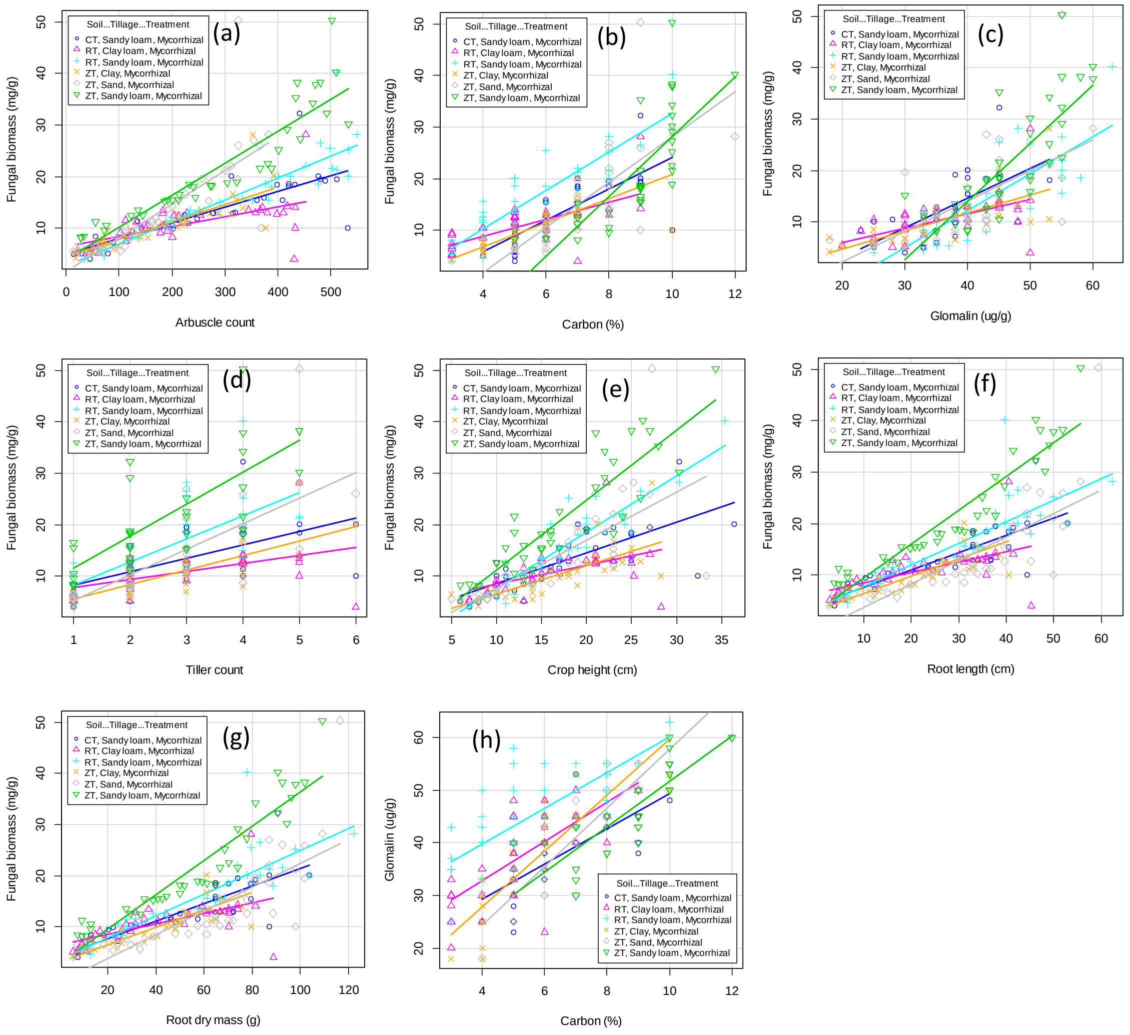

Supplementary figure 3 – Corrected relationships between fungal biomass and (a) arbuscule count, (b) percentage carbon, (c) glomalin, (d) tiller count, (e) crop height, (f) root length, as well as (g) root dry mass in compost soils of 4 soil textures (sandy loam, clay loam, clay and sand), as well as between 3 tillage types (conventional, reduced, and zero). Correlation between soil glomalin and carbon between the mentioned soil textures and tillages can be seen in (h).

Supplementary table 6 – Presented R^2 values for Pearson's correlations of fungal and crop growth attributed shown in Supplementary figure 3.

	Arbuscules count	Carbon (%)	Crop height (cm)	Fungal biomass (mg/g)	Glomalin (ug/g)	Root dry mass (g)	Root length (cm)	Tiller count
Arbuscle count								
Carbon (%)	0.68							
Crop height (cm)	0.84	0.64						
Fungal biomass (mg/g)	0.77	0.75	0.69					
Glomalin (ug/g)	0.77	0.73	0.96	0.72				
Rot dry mass (g)	0.88	0.71	0.85	0.76	0.74			
Root length (cm)	0.88	0.71	0.85	0.76	0.74	1.00		
Tiller count	0.71	0.52	0.55	0.55	0.55	0.74	0.74	
